# Challenges and Insights in the Diagnosis and Management of Orbital Tuberculosis: A Systematic Review of 113 Cases

**DOI:** 10.7759/cureus.68976

**Published:** 2024-09-09

**Authors:** Injam Ibrahim Sulaiman, Mohammed A Bani Saad, Ali A Bani-Saad, Younus M Al-Khazaali, Rania H Al-Taie, Sajjad Al-Badri, Mustafa Ismail

**Affiliations:** 1 Department of Surgery, College of Medicine, Hawler Medical University, Erbil, IRQ; 2 Department of Surgery, College of Medicine, University of Baghdad, Baghdad, IRQ; 3 Department of Surgery, College of Medicine, Al-Nahrain University, Baghdad, IRQ; 4 Department of Surgery, College of Medicine, University of Mustansiriyah, Baghdad, IRQ; 5 Department of Surgery, Baghdad Teaching Hospital, Medical City Complex, Baghdad, IRQ

**Keywords:** antitubercular therapy, diagnostic challenges, extrapulmonary tuberculosis, ocular tuberculosis, orbital tuberculosis

## Abstract

Orbital tuberculosis (O-TB) is an extremely rare manifestation of extra-pulmonary tuberculosis (TB), which affects orbital structures and causes very complex clinical scenarios that may simulate other pathologies affecting the orbit. Its diagnostic and therapeutic challenges are due to its rarity and lack of specificity on symptoms. This systematic review aims to give an in-depth analysis regarding the presentation of clinical features, diagnosis methods, treatment outcomes, and complications, enhancing the current understanding and management of O-TB. A systematic review was conducted in accordance with the Preferred Reporting Items for Systematic Reviews and Meta-Analyses. Literature searches were conducted in PubMed and Scopus up to August 2024. The literature review included case series, case reports, and retrospective studies focusing on O-TB and involved a total of 113 cases from 12 studies. The extracted data were qualitatively synthesized regarding patient demographics, clinical presentations, diagnostic methods, treatment regimens, and the ensuing outcomes.

The review found that O-TB mostly affects subjects with a mean age of 37.75 years, although there is a very wide age range of reported cases: 2-82 years with an almost equal gender distribution. The most common symptoms were vision impairment at 71.68%, eyelid swelling at 9.73%, and exophthalmos at 5.31%. Imaging, especially with computed tomography (CT) scans in 60.18% of patients, along with histopathological confirmation and molecular biological confirmation positive in 46.02%, was the principal tool for diagnosis. In most cases, antitubercular therapy (ATT) was the mainstay of treatment, leading to complete resolution. However, 30.09% of these cases had some complications like glaucoma and cataracts that point to careful management and follow-up. O-TB still remains a diagnostic challenge due to its rarity and changing clinical presentations. Early diagnosis and identification, presently mainly through imaging and histopathological examination, is important for the management. This review shows the effectiveness of ATT in the treatment of O-TB but also brings out the need for better diagnostic facilities and uniformity in treatment protocols so that complications can be prevented and outcomes improved. Future studies must be directed toward the development of more sensitive diagnostic tools and the elucidation of immune responses in O-TB guiding better clinical practice.

## Introduction and background

Orbital tuberculosis (O-TB) though a rare manifestation of TB, represents a significant diagnostic and therapeutic challenge due to its ability to mimic various other orbital conditions such as neoplasms, inflammatory pseudotumors, and abscesses. This mimicry often leads to delays in diagnosis, which can have profound consequences on patient outcomes, including visual impairment and life-threatening complications [1].

The global incidence of O-TB is closely tied to the prevalence of TB, with higher rates observed in endemic regions such as South Asia and parts of Africa [2]. However, increasing rates of extrapulmonary TB, including O-TB, have also been reported in developed countries, driven by factors such as immigration, immunosuppression, and the resurgence of TB in specific populations [3]. Clinically, O-TB presents with a wide spectrum of symptoms, including proptosis, eyelid swelling, diplopia, pain, and visual impairment, all of which vary depending on the extent of orbital involvement [1]. Imaging, particularly CT and magnetic resonance imaging (MRI), plays a crucial role in the diagnosis, revealing characteristic findings such as soft tissue masses, bony erosions, and orbital abscesses. However, definitive diagnosis relies on microbiological and histopathological confirmation, often involving polymerase chain reaction (PCR) testing and acid-fast bacilli (AFB) staining [2]. Despite the challenges in diagnosis, treatment outcomes for O-TB are generally favorable when managed appropriately with antitubercular therapy, which typically includes a combination of isoniazid, rifampicin, pyrazinamide, and ethambutol. The duration of treatment is often prolonged, ranging from six months to a year, depending on the severity of the disease and the patient’s response to therapy [2]. However, challenges such as drug resistance and the need for surgical intervention in complex cases underscore the importance of timely diagnosis and a multidisciplinary approach to management.

This paper aims to synthesize the current literature on O-TB, focusing on clinical presentations, diagnostic strategies, therapeutic interventions, and long-term outcomes. By addressing the challenges and best practices in the management of this rare but significant condition, we want to provide insights that will enhance the care of patients with O-TB and improve overall treatment outcomes.

## Review

Methods

Study Design

This systematic review adhered to the guidelines outlined by the Preferred Reporting Items for Systematic Reviews and Meta-Analyses (PRISMA) [4]. The aim was to comprehensively review innovations in surgical techniques and adjuvants used in trabeculectomy. Ethical review board approval or informed consent from participants was not required due to the nature of the study.

Literature Search and Study Selection

A comprehensive literature search was conducted by two independent investigators across two databases, including PubMed and Scopus, up to August 8, 2024. The search terms included "orbital tuberculosis," "ocular tuberculosis," "extrapulmonary tuberculosis," and "orbital inflammation." Articles were selected based on their relevance to the subject, particularly those focusing on clinical presentations, diagnostic strategies, treatment outcomes, and complications.

**Figure 1:**
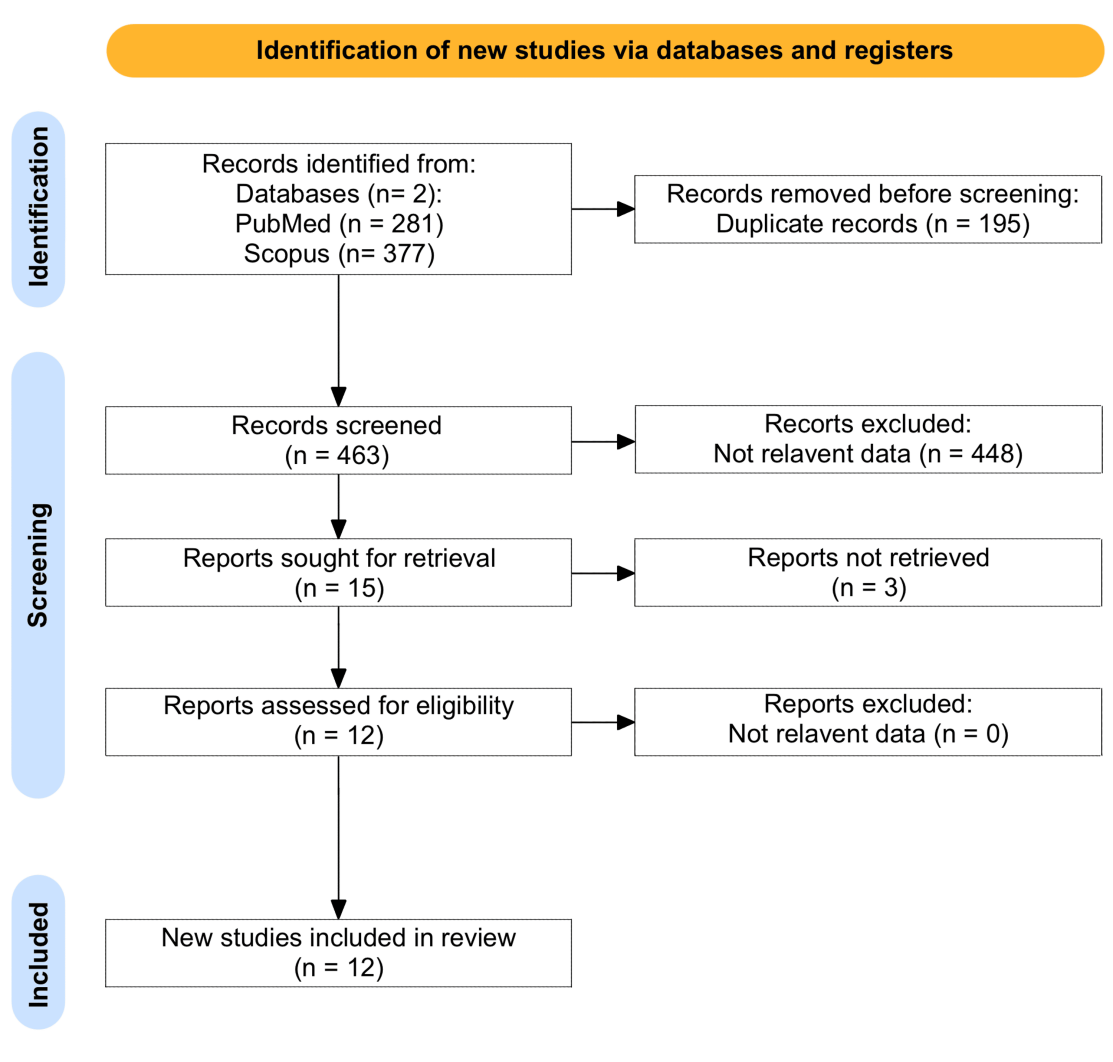
PRISMA flow chart PRISMA: Preferred Reporting Items for Systematic Reviews and Meta-Analyses

The study selection process is detailed in Figure 1. The initial search yielded a total of 658 studies. After removing duplicates, the titles and abstracts of 463 studies were screened for relevance. Inclusion criteria were as follows: studies that focused on O-TB reported primary data on clinical presentation, diagnosis, treatment, and outcomes, and were published in peer-reviewed journals. Both case reports and case series were included to capture the full spectrum of clinical presentations and outcomes. Exclusion criteria included studies that did not focus specifically on O-TB, those without sufficient clinical data, and reviews or editorials that did not present new data. After the screening process, 15 articles were eligible for the systematic review, 3 of which were not retrieved so 12 studies were included in the systematic review. These studies comprised six case series, five case reports, and one review article, totaling 113 cases of O-TB.

Inclusion and Exclusion Criteria

Inclusion criteria consisted of studies focusing on O-TB, studies published in English, and full-text articles providing detailed descriptions of clinical presentations, diagnostic methods, therapeutic interventions, and outcomes. Exclusion criteria consisted of non-English publications, irrelevant articles (such as reviews or abstracts), retracted articles, and studies not directly related to O-TB.

Data Extraction

Data were extracted from the selected studies using a standardized data extraction form. The extracted data included study characteristics (author, year of publication, country of origin, study type), patient demographics (age, gender, HIV status), clinical presentation (symptoms, duration of symptoms), diagnostic methods (imaging, laboratory tests, histopathological findings), treatment details (antitubercular therapy, surgical interventions), outcomes (response to treatment, follow-up duration, complications), and case fatality rates. Particular attention was paid to the variety of clinical presentations and the effectiveness of different diagnostic and therapeutic approaches. The extracted data were independently reviewed by two investigators to ensure accuracy and consistency.

Data Synthesis and Quality Assessment

The extracted data were synthesized qualitatively, given the heterogeneous nature of the included studies. Descriptive statistics were used to summarize the patient demographics, clinical features, diagnostic methods, and treatment outcomes. Each selected article underwent a qualitative assessment to evaluate its contribution to the field, focusing on methodology, results, and conclusions. Cross-referencing citations within these articles helped identify additional relevant studies, ensuring a comprehensive review of the subject matter.

The ROBINS-I was employed to evaluate the quality of the cohort studies that were included [5]. Studies were classified as low, moderate, or high quality based on their scores.

Results

Study Characteristics and Patient Demographics

This systematic review includes a total of 12 studies, including 113 cases of O-TB. These were six case series, five case reports, and one review article spanning the years 1994-2021. They were conducted in different countries - India, the United Kingdom, Malaysia, Australia, and China - to represent the trends for O-TB cases worldwide.

Table 1 provides information pertaining to each study [6-17]. In this table, the results of a variety of studies are presented that demonstrate the efficacy, safety, and clinical outcomes regarding the techniques and adjuvants. Furthermore, Table 2 represents the results of the risk assessment bias which exhibited an overall low risk.

**Table 1:** A comprehensive overview of the included articles ESR: erythrocyte sedimentation rate; PCR: polymerase chain reaction; AFB: acid-fast bacilli; MTB: *Mycobacterium tuberculosis*; FFA: fundus fluorescein angiography; ICGA: indocyanine green angiography; OCT: optical coherence tomography; TB: tuberculosis; ATT: antitubercular therapy; TST: tuberculin skin test; FNAC: fine needle aspiration cytology; BCVA: best corrected visual acuity; PPD: purified protein derivative

Study identifier and publication year	Study type	Timeframe of the study	Sample size	Country	Age (mean, SD)	Gender distribution	HIV status	Symptoms	Duration of symptoms (mean, SD)	Imaging	Laboratory tests	Histopathological findings	Antitubercular Therapy (ATT)	Surgical Intervention	Drug-Resistant TB	Response to Treatment	Follow-up Duration	Case Fatality Rate	Complications
Babu et al. 2014 [6]	Case series	2010-2013	6 cases	India	Mean 38.0 ± 14.85 years	5 females (83.3%); 1 male (16.7%)	Not specified	Swelling, periostitis, bone involvement, dacryoadenitis	Mean 2.33 ± 1.21 months	CT scan used for diagnosis	Positive Mantoux test, ESR, PCR, Histopathology	Granulomatous inflammation with caseation	Isoniazid, Rifampicin, Ethambutol, Pyrazinamide	Biopsy, abscess drainage	Not reported	Complete recovery in most cases	Mean 9.75 months	None	One case with inadequate treatment history
Bajaj et al. 2016 [7]	Case report	Not specified	2 cases	India	Mean 40.5 ± 28.99 years	2 females (100.0%)	Not specified	Eyelid swelling, discharging sinus	Mean 3.5 ± 0.71 months	None	Mantoux test, AFB smear	Granulomatous inflammation with caseation	Rifampicin, Isoniazid, Ethambutol, Pyrazinamide	None	Not reported	Complete recovery	9 months	None	None
Biswas et al. 2016 [8]	Retrospective study	2011-2016	40 cases	India	Mean 40.6 ± 18.05 years	15 females (37.5%); 25 males (62.5%)	Not specified	Choroiditis, vision impairment	Not specified	Fundus photography, FFA	Positive Mantoux test, ESR	Positive for MTB DNA	Rifampicin, Isoniazid, Ethambutol, Pyrazinamide	None	Not reported	Resolution of symptoms	9 months	None	None
Gupta et al. 2015 [9]	Review article	Not applicable	Not applicable	Multi-country	Not applicable	Not applicable	Not specified	Various ocular symptoms including anterior uveitis, intermediate uveitis, posterior uveitis, scleritis, choroidal lesions	Not applicable	Fundus photography, FFA, ICGA, OCT	PCR for MTB, histopathology, tuberculin skin test, interferon-gamma release assay	Granulomatous inflammation, choroidal tubercles, scleritis, vasculitis	Rifampicin, Isoniazid, Ethambutol, Pyrazinamide	Not applicable	Not reported	Varied depending on the case	Not specified	None	None
Hughes et al. 2008 [10]	Case series	1975-2006	7 cases	UK	Mean 34.0 ± 10.0 years	5 females (71.4%); 2 males (28.6%)	All HIV-negative	Visual loss, ocular pain, constitutional symptoms	Mean 25.5 ± 34.65 weeks	MRI abnormalities in 6 out of 7 patients	Positive Mantoux test, elevated ESR in 2 patients	Granulomatous inflammation in lymph node biopsies of 2 patients	Rifampicin, Isoniazid, Pyrazinamide, Moxifloxacin	None	Not reported	Improvement in visual acuity in all cases	Mean 38.9 weeks	None	Steroid-related complications in one patient
Khurana et al. 2014 [11]	Case series	2009-2012	8 cases	India	Mean 10.75 ± 5.74 years	5 females (62.5%); 3 males (37.5%)	Not specified	Discharging sinus, cystic mass, lid necrosis	Mean 12.25 ± 16.62 weeks	CT scan findings varied based on presentation	Positive PCR in 4 cases, positive AFB culture in 3 cases	Granulomatous inflammation in 6 cases, caseous necrosis in 1 case	Isoniazid, Rifampicin, Pyrazinamide, Ethambutol (2 months), followed by Isoniazid, Rifampicin (4 months)	Needle aspiration, drainage and curettage, sinus debridement, tissue debridement, skin grafting, lid reconstruction	Not reported	Complete resolution in all cases	Not specified	None	Reconstructive surgery needed in 3 cases
Mittal et al. 2017 [12]	Retrospective case series	2012-2013	6 cases	India	Mean 37.75 ± 19.19 years	3 females (50.0%); 3 males (50.0%)	All immunocompetent and HIV-negative	Pain, redness, swelling, orbital mass	Mean 1.25 ± 1.06 months	CT showed soft tissue mass in all cases	Positive PCR in all cases, ESR elevated in 3 cases, TST positive in 3 cases	Granulomatous inflammation in 5 cases, caseous necrosis in 5 cases	Isoniazid, Rifampicin, Pyrazinamide, Ethambutol	Biopsy performed in all cases	Not reported	Complete resolution in 5 cases, partial resolution in 1 case	Mean 11 months	None	None
Patkar et al. 1994 [13]	Case report	Not specified	3 cases	India	Mean 12.0 ± 2.0 years	3 females (100.0%)	Not specified	Proptosis, diplopia, focal seizure (case 1)	Mean 5.0 ± 4.24 weeks	CT showed hyperdense lesion in all cases	Positive Mantoux test, ESR raised, FNAC positive for AFB (case 1), negative for AFB (case 2)	Granulomatous inflammation in all cases, caseous necrosis in 2 cases	Streptomycin, Isoniazid, Rifampicin, Pyrazinamide (2 months) followed by Isoniazid, Rifampicin (18 months)	Transcranial exploration, lateral orbitotomy, needle biopsy	Not reported	Complete recovery in all cases	Mean 5.7 months	None	None reported
Shahidatul-Adha et al. 2017 [14]	Retrospective case series	2011-2016	34 cases	Malaysia	Mean 45.5 ± 51.62 years	15 females (44.1%); 19 males (55.9%)	1 patient with HIV infection	Blurred vision, eye redness, discomfort, photophobia, tearing	Mean 3.13 ± 4.07 months	Normal chest radiography in 79.4% of cases	Positive Mantoux test in 94.1% of cases, raised ESR in 58.8% of cases	Non-caseating granuloma in 1 case	Ethambutol, isoniazid, rifampicin, pyrazinamide for 6–12 months	Cataract surgery, filtering surgery for glaucoma	Not reported	Variable visual outcome, BCVA equal or better than 6/60 in 62.5% of eyes at 1-year follow-up	Duration varied from 6 to 12 months	3 deaths reported, unrelated to ocular TB	Glaucoma, cystoid macular edema, cataract, macular scar, retinal vein occlusion
Sodhi et al. 2004 [15]	Case report	Not specified	2 cases	India	Mean 2.5 ± 0.71 years	1 female (50.0%); 1 male (50.0%)	Not Specified	Proptosis, fever, orbital swelling, sinus discharge	Mean 2.5 ± 0.71 months	CT scan (orbital mass, bony destruction)	Positive Mantoux test, ESR raised, FNAC showed caseous necrosis with AFB in one case, culture negative in another	Caseous necrosis, AFB in one case	Isoniazid, Rifampicin, Ethambutol, Pyrazinamide	None	Not reported	Complete recovery in both cases	Not specified	None reported	None reported
Yao et al. 2018 [16]	Case report	January 1, 1994-December 12, 2016	3 cases	Australia	Mean 51.5 ± 10.61 years	1 female (33.3%); 2 males (66.7%)	Not Specified	Dacryoadenitis, proptosis, lid swelling, night sweats, diplopia, globe displacement, cervical lymphadenopathy	Mean 0.625 ± 0.18 months	CT scan (orbital mass, lacrimal gland swelling, infiltrative soft tissue mass)	Positive Mantoux test, culture positive for *Mycobacterium tuberculosis* in one case, necrotizing granulomatous inflammation, acid-fast bacilli detected in one case	Granulomas with necrotizing granulomatous inflammation, multinucleated giant cells, caseous necrosis	Isoniazid, Rifampicin, Pyrazinamide, Ethambutol	Biopsy for diagnosis	Not reported	Clinical improvement in all cases, complete resolution in some cases	Treatment ongoing for one case, others completed 9-month regimen	None reported	Visual loss, bony destruction, intracranial extension (potential complications mentioned)
Zhao et al. 2021 [17]	Case report	Not specified	2 Cases	China	Mean 40.5 ± 2.12 years	2 females (100.0%)	Not Specified	Orbital mass, palpebral swelling, no significant pain, no visual impairment	Mean 2.0 ± 1.41 months	CT scan (irregular soft tissue densities in the lacrimal gland area bilaterally)	Positive PPD skin test for tuberculosis, normal chest X-ray, erythrocyte sedimentation rate normal	Granulomatous inflammation with caseation, giant cells, fibrous tissue proliferation	Isoniazid, Rifampicin, Ethambutol, Pyrazinamide	Excision biopsy of the orbital masses	Not reported	Complete resolution of the masses without residuals after 1 year of ATT	1 year	None reported	None reported

**Table 2:** Risk-of-bias assessment of the included studies ROBINS-I tool is used to assess the risk of bias.

Authors	Confounding	Selection of patients	Classification of interventions	Deviations from intended interventions	Missing data	Measurement of outcomes	Selection of reported results
Patkar et al. 1994 [13]	Moderate	Moderate	Low	Low	Moderate	Moderate	Low
Sodhi et al. 2004 [15]	Moderate	Serious	Low	Low	Serious	Serious	Moderate
Hughes et al. 2008 [10]	Moderate	Low	Low	Low	Low	Low	Low
Babu et al. 2014 [6]	Moderate	Moderate	Low	Low	Moderate	Moderate	Low
Khurana et al. 2014 [11]	Moderate	Low	Low	Low	Low	Moderate	Moderate
Gupta et al. 2015 [9]	Moderate	Moderate	Low	Low	Low	Moderate	Moderate
Biswas et al. 2016 [8]	Moderate	Moderate	Low	Low	Low	Moderate	Low
Bajaj et al. 2016 [7]	Moderate	Low	Low	Low	Low	Low	Low
Shahidatul-Adha et al. 2017 [14]	Moderate	Moderate	Low	Low	Low	Moderate	Low
Mittal et al. 2017 [12]	Moderate	Moderate	Low	Low	Moderate	Low	Low
Yao et al. 2018 [16]	Moderate	Moderate	Low	Low	Low	Moderate	Low
Zhao et al. 2021 [17]	Moderate	Low	Moderate	Low	Low	Moderate	Low

**Figure 2:**
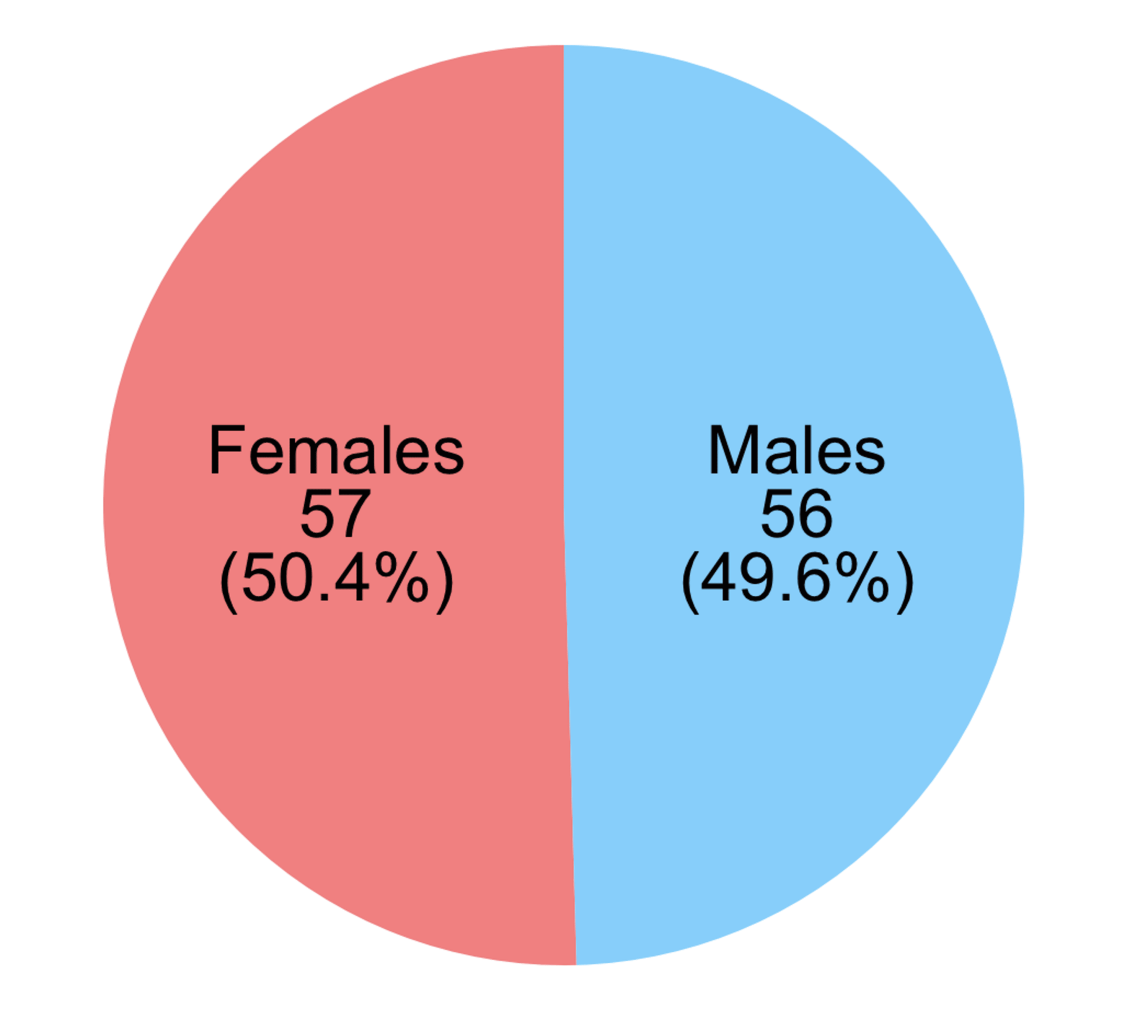
Gender distribution in orbital tuberculosis cases

**Figure 3:**
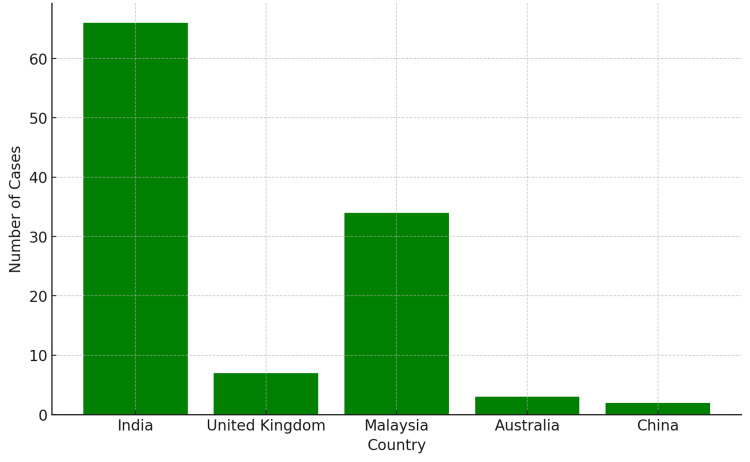
Number of patients from each country

The range of ages was found to be from 2 to 82 years, with an average of 37.75 years for all studies. There were both pediatric and adult populations of the cases reported, with remarkable variability within the distribution of age ranges. For instance, Khurana et al. (2014) [11] reported in 2014, the age range of the patients in theirs was between 3 and 16 years. In this study, they focused purely on pediatrics. In contrast, the study by Shahidatul-Adha et al. (2017) [14] included a broader age range, from 9 to 82 years, reflecting the potential for O-TB to affect individuals across the lifespan. Gender-wise pretty equal, the ratio of the cases is 50.4:49.6% in females to males. A more considerable trend is like the studies of Babu et al. (2014) [6] and Khurana et al. (2014) [11], where females were 83% and 62.5%, respectively, of the cases. However, other studies, like Biswas et al. (2016) [8], reported a higher male-to-female ratio with 62.5% of the cases being male (Table 3, Figures 2-3).

**Table 3:** Demographic data of the studies overall

Category		Data
Total cases		113
Mean age (range)		Mean 37.75 ± 19.19 years (2-82)
Sex	Females	57 (50.4%)
	Males	56 (49.6%)
Study types	Case series	6
	Case reports	5
	Review articles	1
Countries included		India, UK, Malaysia, Australia, and China
HIV status reported	Positive	1
	Negative	15
	Status not reported	97

HIV status was not generally reported or tested for, apart from in Hughes et al. (2008) [10], where all patients were confirmed to be HIV-negative, and Shahidatul-Adha et al.'s study (2017) [14], in which one patient was reported to be HIV-positive.

Clinical presentation

**Figure 4:**
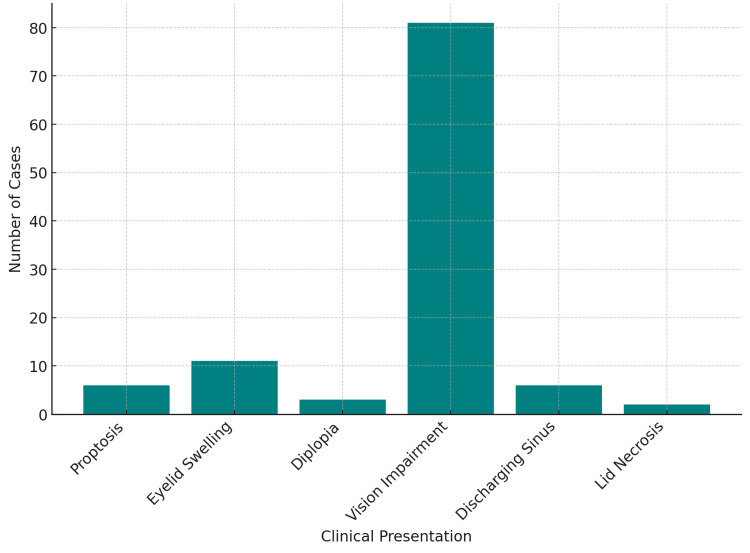
Clinical presentations in orbital tuberculosis cases

**Figure 5:**
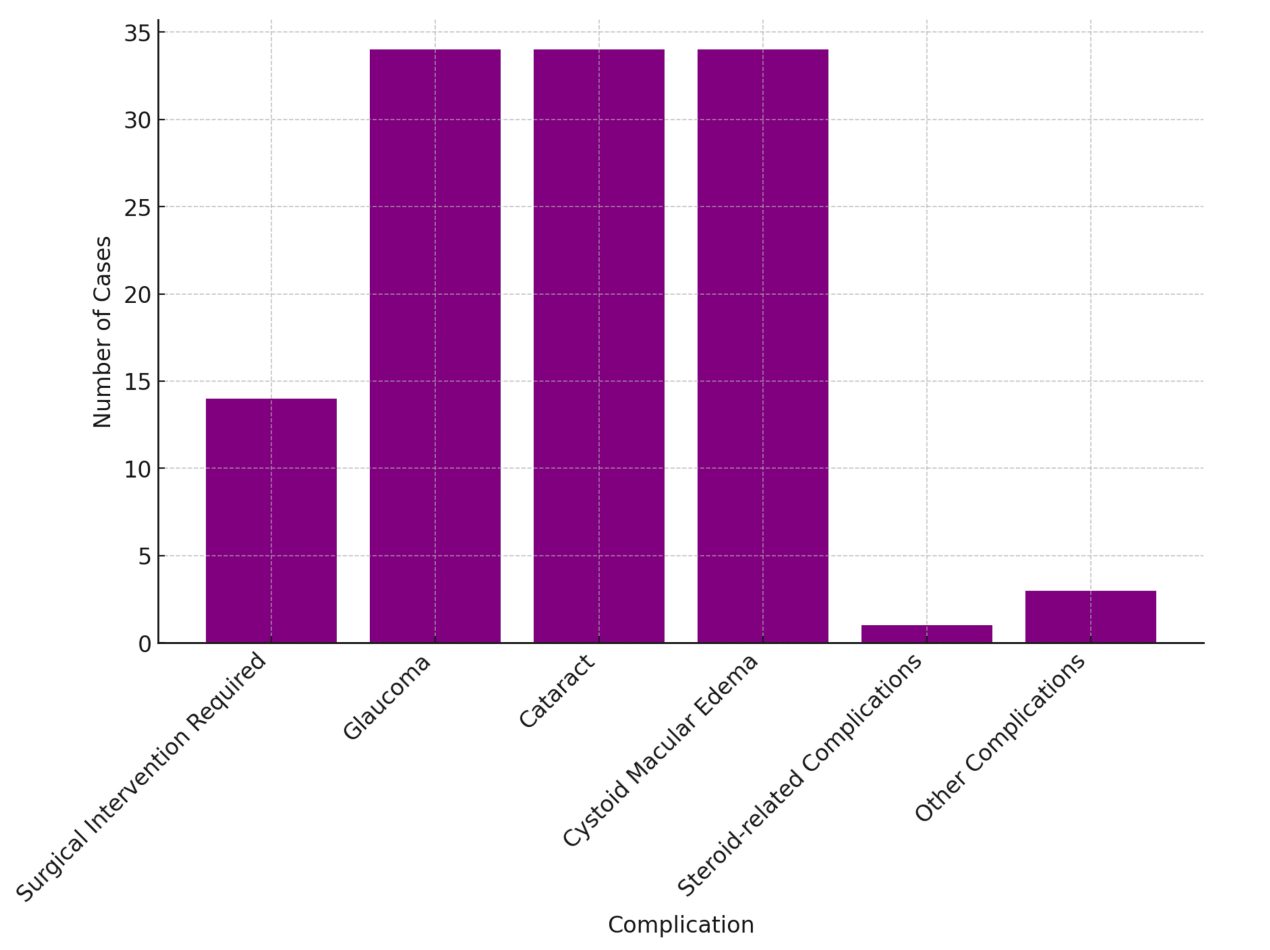
Complications in orbital tuberculosis cases

A summary of the clinical data is emphasized in Table 4 and Figures 4-5. The clinical presentation of O-TB was highly variable, reflecting the potential of the disease to involve different structures within the orbit. Prominent common symptoms were proptosis with swelling of the eyelid, diplopia, and loss of vision. Specifically, proptosis was observed in six cases (5.31%), particularly when coupled with the symptom of diplopia in three of these cases (2.65%) and sometimes with pain, characteristics similar to those exhibited in the study by Patkar et al. (1994) [13] and Sodhi et al. (2004) [15]. Eyelid swelling was differentially observed in 11 cases (9.73%). The most common presenting complaint was visual dysfunction, present in 81 cases (71.68%).

**Table 4:** Clinical summary of the studies overall ATT: antitubercular therapy; PCR: polymerase chain reaction; AFB: acid-fast bacilli

Category		Number (percentage)
Clinical presentation	Proptosis	6 cases (5.31%)
	Eyelid swelling	11 cases (9.73%)
	Diplopia	3 cases (2.65%)
	Vision impairment	81 cases (71.68%)
	Discharging sinus	6 cases (5.31%)
	Lid necrosis	2 cases (1.77%)
Complications	Complete resolution with ATT	109 cases (96.46%)
	Surgical intervention required	14 cases (12.39%)
	Glaucoma	34 cases (30.09%)
	Cataract	34 cases (30.09%)
	Cystoid macular edema	34 cases (30.09%)
	Steroid-related complications	1 case (0.88%)
	Other complications	3 cases (2.65%)
Diagnostic methods used	CT scan	68 cases (60.18%)
	MRI	7 cases (6.19%)
	Positive Mantoux test	104 cases (92.04%)
	Positive PCR for MTB	52 cases (46.02%)
	Histopathology (granulomatous inflammation)	113 cases (100%)
	AFB staining positive	9 cases (7.96%)
Treatment	Antitubercular therapy (ATT)	113 cases (100%)
	Surgical intervention (e.g., abscess drainage, biopsy)	14 cases (12.39%)
	Reconstructive surgery	3 cases (2.65%)

In another study, Khurana et al. (2014) [11] demonstrated others involving discharging sinus, cystic masses, and lid necrosis, in which six cases (5.31%) had a discharging sinus and two cases (1.77%) of lid necrosis with a link to pediatric patients. The duration of symptoms noted before a definitive diagnosis ranged from less than two weeks to those greater than six months across all studies. For Khurana et al., the median duration was 12.25 weeks, and reported by Hughes et al. was 25.5 weeks, the average duration of the symptoms before diagnosis. This variation in the duration of symptoms highlights one of the multiple difficulties in the diagnosis of O-TB, which is most often insidious in its presentation and mimics other orbital conditions.

Diagnostic methods

Imaging modalities played a major role in the diagnosis of O-TB. However, CT imaging was very commonly used in studies and was done in 68 cases (60.18%). The common findings on CT imaging were hyperdense lesions, soft tissue mass, or bony erosions suggesting tubercular involvement. Examples are that of Sodhi et al. (2004) [15], where CT scans revealed massive bony destruction of the orbital walls due to his disease, thus reflecting the aggressive nature of the disease in some patients; on the other hand, MRI was infrequently used but provided good information in those cases with predominant soft tissue involvement, as well noted by Hughes et al. Laboratory investigations, such as the Mantoux test and ESR, were frequently performed to aid in the diagnosis. The Mantoux test was positive in 104 cases (92.04%) in the multiple studies according to Shahidatul-Adha et al.(2017) [14], where a positive Mantoux test was found in 94.1% of the cases. High ESR was another observation, more so in those with diffuse systemic involvement. A definite diagnosis was based on microbiological and histopathological confirmation. The PCR for *Mycobacterium tuberculosis* DNA was positive in 52 cases in various studies, including that by Biswas et al. in 2016, where PCR detected MTB DNA in 57.14% of cases. Histopathological examination mostly returned granulomatous inflammation with caseation necrosis, similar to most of the cases in both studies by Patkar et al. [13] and more than two decades ago by Zhao et al. (2021) [17] AFB was less often diagnosed, and AFB positive stains were only reported in nine cases (7.96%) as described by Mittal et al. (2017) [12].

Treatment and outcomes

ATT was the cornerstone of treatment in all studies, and the standard regimen used was isoniazid, rifampicin, pyrazinamide, and ethambutol. The duration of ATT varied as per the study and the severity of the disease, ranging for a period of 6-12 months. In most cases, ATT was rather successful in causing resolution of symptoms and preventing recurrence. For instance, Mittal et al. (2017) [11] described complete resolution in 83.3% of their cases and only one case had a partial resolution, whereas Khurana et al. (2014) [11] described complete resolution of all cases post-ATT and surgical intervention.

In 14 cases, abscess formation or extensive tissue destruction required therapeutic surgery or a biopsy was necessary for diagnosis. The common surgical procedures are drainage of abscess, biopsy, and debridement, as described in the case series by Khurana et al. (2014) [11] and Babu et al. (2014) [6]. Three cases had extensive tissue loss, mainly in the pediatric age group, which had to be managed with reconstructive surgery. This response to treatment was overall good in most cases, with complete recovery and resolution of symptoms stated by most studies; however, several studies noted complications in some of their patients in Hughes et al. (2008) [10], steroid-related complications were described in one patient (0.88%), and Khurana et al. (2014) [11] reported reconstructive surgery was required in three cases due to corporal rupture.

There was no report of tuberculosis (TB) resistance from the review of studies and the case fatality rate was rather low; Shahidatul-Adha et al.(2017) [14] reported three deaths completely unrelated to ocular TB.

Complications and follow-up

The complications related to O-TB were few in number, relative to the benign nature of the pathology, but when present were mostly an extension of the severity of the disease process and the amount of tissue involved. For example, Shahidatul-Adha et al. (2017) [14] have described glaucoma, cystoid macular edema, and cataract; each in 34 cases (30.09%) in their series. Like them, Sodhi et al. [15] cited proptosis and restriction of ocular movements due to diffuse orbital involvement. There was a wide variation between studies in the duration of follow-up, from those with detailed long-term follow-up data to those with just short-term outcomes. For example, Hughes et al. (2008) [10] reported that the average duration of follow-up was 38.9 weeks, in which for all patients, visual activity was recovering. A 2014 article by Babu et al. (2014) [6] found that cases had an average duration of 9.75 months, with most of the cases recovering completely.

Summary

Results of the present systematic review underline the different patterns of clinical presentations and outcomes in O-TB. While ATT forms the mainstay of treatment, timing at presentation often influences treatment; therefore, early diagnosis and proper surgical intervention are important in preventing complications and ensuring a favorable outcome. There is thus a need to maintain a high index of suspicion regarding O-TB, especially in endemic regions, so as to ensure prompt diagnosis and management.

Discussion

O-TB is a rare manifestation of extrapulmonary tuberculosis, particularly even in regions where TB is endemic, such as India [2]. Cases of over 10 million occur annually across the world, with the largest burdens recorded in countries such as India, China, and South Africa [3]. Given this global burden, O-TB is grossly underreported because of the difficulties in making a diagnosis and its ability to mimic other diseases that often delay its diagnosis. It occurs more in children, particularly in regions where it is endemic and is frequently associated with HIV and drug-resistant TB strains. In these areas, O-TB therefore has to be kept invariably in the differentials for such unexplained orbital masses, especially when initial treatments fail [18]. This systematic review illustrates the challenges in the diagnosis and management of O-TB, which is a rare but important variety of extrapulmonary tuberculosis. It involved an analysis of 113 case reports from 12 studies regarding clinical presentation, methods of diagnosis, treatment outcomes, and complications.

The demographic analysis of O-TB cases showed an almost equal distribution in both genders. However, some studies, like that by Babu et al. (2014) [6], have reported a female predominance, and others, like Biswas et al. (2016) [8], higher incidences in the male gender. There is a disparity by gender, and this may be explained by underlying risks, sociocultural factors, or probably some rather unknown vulnerabilities specific to the gender. The wide age range is equally significant, from 2 to 82 years. The pediatric cases are mostly very symptomatic, in particular those reported by Khurana et al. in 2014 [11], with presentation including discharging sinuses and lid necrosis. The place of geographic location is worldwide in spread but has the greatest prevalence in TB-endemic regions, which include India, the United Kingdom, Malaysia, Australia, and China. That is to say, immigration and travel from these endemic regions are crucial in creating the occurrence of O-TB cases in non-endemic countries, indicating increased awareness among healthcare providers who can make a diagnosis in non-endemic regions. Furthermore, results from Zhao et al. (2021) [17] and Yao et al. (2018) [16] further underline that immigration and travel from endemic regions are important determinants for the occurrence of O-TB in a non-endemic country. The results from these authors about their study lead to the need to increase awareness of this entity for healthcare providers.

Clinical presentations and diagnostic challenges

The clinical presentation in O-TB is remarkably polymorphic, including all orbital structures, hence complicating diagnosis. Among the prevailing symptoms of this disease, visual impairment, proptosis, eyelid swelling, and diplopia are the most frequent; among these, vision impairment is the most frequent and serious symptom. Another confusing feature while diagnosing is that it is a great mimicker of other conditions in the orbit, like neoplasms or inflammatory pseudotumors. For example, Patkar et al. (1994) [13] and Khurana et al. (2014) [11] reported cases of O-TB presenting with quite nonspecific symptoms, so they could have been easily misdiagnosed. The duration of symptoms was significantly longer before diagnosis - up to a median of 25.5 weeks in some cases - emphasizing the role of heightened clinical suspicion, especially in endemic regions or among patients with a history of TB. This review emphasizes that the median duration of symptoms before diagnosis ranged from some weeks to months, with some cases extending to 25.5 weeks of duration as observed by Hughes et al. in 2008 [10]. It has been suggested that this underlines the importance of heightened clinical suspicion, but if it is an endemic area or there is a history of tuberculosis, then during such a nonspecific and rare condition, there may be a diagnosis.

Makhoba et al. in 2020 [19] identified some key biomarkers that help the immune response to O-TB: a four-marker biosignature-CD40L, IL-33, IFN-γ, and SAP. These biomarkers bear relevance for the diagnosis of O-TB, especially in areas where HIV infection forms the background of high prevalence; therefore, it has an important role in the progression of disease-based immune response. The understanding of immune responses refines diagnostic approaches and brings guidelines onto treatment strategies, more so for immunocompromised patients.

Diagnostic modalities and their efficacy

Imaging, laboratory, and histopathological findings are all used for the diagnosis of O-TB. According to the review, CT was used in 60.18% of cases, mostly with the appearance of hyperdense lesions, soft tissue mass, or bony erosions that suggested tubercular involvement. The value of MRI lies in its use with predominating soft tissue involvement. The work of Hughes et al. (2008) [10] and Sodhi et al. (2004) [15] demonstrated that CT and MRI are both useful for the accurate delineation of the extent of orbital involvement, particularly in such complex cases with suspected bone destruction. Imaging modalities are sure to have an important role in diagnosing and managing O-TB due to its rarity. Bone destruction and abscesses extending into the local vicinity will be best picked up by CT. However, the best way to assess soft tissue involvement, like the lacrimal gland and adjacent brain structures, is by MRI, giving better details without bony artifacts. Thus, a combination of CT and MRI certainly provides a comprehensive assessment, allowing an enhanced diagnosis and treatment plan for the better management of patient outcomes [20].

Laboratory diagnostics - including Mantoux testing and PCR for *M. tuberculosis* DNA - were carried out to confirm the diagnosis; PCR turned out positive in 46.02%. AFB detection by staining was very low, at 7.96%, pointing to microbiological confirmation difficulties in paucibacillary samples. Imaging plays an important role in the differential diagnosis of O-TB from these diseases. In contrast to most orbital tumors, erosive or osteomyelitic change typified O-TB, which is rarely seen in most orbital tumors. Caseating granulomas and AFB are also specifically present in the biopsy material of O-TB, unlike conditions like sarcoidosis which are characterized by non-caseating granulomas. An accurate diagnosis is important because treatment for O-TB involves long-term antituberculous therapy, significantly different from treatments for other orbital conditions. Inappropriate management in the form of unnecessary surgery or inappropriate medication would ensue if there is a misdiagnosis, quite possibly precipitating a deterioration in the patient's condition [1].

Treatment outcomes and complications

ATT was used in all studies included in this review, mostly with a regimen of isoniazid, rifampicin, pyrazinamide, and ethambutol. Treatment duration varied from six months to 12 months and, in most, the outcome was favorable since most of the patients showed complete resolution of symptoms. Complications like glaucoma, cataracts, and cystoid macular edema developed in 30.09% of cases, so even after treatment with ATT, continuous monitoring in these patients is a must. Surgical interventions were rarely required but were effective in cases where complications like abscesses or large tissue destruction developed. For instance, Khurana et al. (2014) [11] reported the pediatric patient's need for reconstructive surgery, highlighting that O-TB may become the cause of gross tissue destruction, particularly when the diagnosis and treatment are delayed.

O-TB can result in serious complications such as glaucoma, cataracts, and cystoid macular edema. Each of them affects about 30% of cases Shahidatul-Adha et al. (2017) [14]. Hence, close follow-up is warranted among patients on ATT, more so if pre-existing known ocular conditions or treatment courses are more prolonged. Surgical interventions, though infrequent, are essential where complications such as abscesses or diffused tissue damage occur. Procedures like drainage of abscesses, biopsy, and debridement are important in managing these complications and the process of healing. The literature suggests that with appropriate treatment of orbital and adnexal tuberculosis in non-HIV-infected patients, generally a good result is obtained in the long term [21]. In our follow-up, there were no recurrences in Directly Observed Treatment (DOTS) category I-ATT-treated patients with a pretty high cure rate of 96.7% in this study, after a follow-up of five years. Complications that required surgical intervention were rare, and for most patients, surgical interventions showed a marked improvement in the quality of life after treatment.

Challenges and future directions

Management of O-TB is problematic in view of the lack of standardized treatment protocols. As Zhang et al. (2023) [22] further accentuated ''The diagnosis of osteoarticular TB is inherently problematic to the paucibacillary nature and therefore delayed or presumptive treatment'.' Marked variability in clinical presentation makes it even more challenging to define a uniform treatment algorithm. Furthermore, the emergence of drug-resistant strains of TB poses a significant challenge to effective management." Such future research must focus on more precise diagnostic tools and biomarkers to achieve treatment tailored to the patient, taking into account fundamental heterogeneity in disease manifestation and drug response. Treatment for O-TB, until quite recently, often needed a multidisciplinary approach to manage such complex cases. OT-B generally gets misdiagnosed due to its potential to masquerade for any other orbital disease like cellulitis, neoplasm, or sarcoidosis. The paper by Madge et al. (2008) [1] focuses more on the fact that O-TB can manifest on X-rays like periostitis, soft tissue tuberculoma, or cold abscess, making it look like a host of other things. Thus, for example, in O-TB, periostitis may have a clinical resemblance to orbital cellulitis. However, it is less acute and may be extended over a longer duration. This heralds bone destruction, which is more like what we get in O-TB.

This, as shown in Baluku et al. (2021) [23], requires an immediately expanded team that includes professionals from ophthalmologists, infectious disease experts, radiologists, and surgeons. All these specialists make it possible to carry out individual methods of treatment, especially when it is difficult to act under standards either because of drug resistance or the presence of comorbidities. This collaborative realization of different sub-specialties allows better treatment success rates and improved quality of life through the attention to ocular and systemic aspects related to the disease.

Magombedze et al. (2021) [24] put much emphasis on the role of bacterial load slopes as a biomarker for the prediction of success, failure, and relapse during the therapy of TB. These biomarkers were particularly sensitive and specific for the γ_s slope in the prediction of a relapse-free cure. Such a biomarker, as part of management in O-TB, would identify those at risk of relapsing early enough to inform the duration of therapy and closer, long-term monitoring. Such biomarkers should be tested on a regular basis so that treatment effectiveness can be assessed, which reduces the risk of many complications due to relapse through orbital involvement.

Implications for clinical practice and future research

This review further highlights the critical clinical implications for the management of O-TB and raises awareness regarding endemic areas or patients with a history of tuberculosis. It further suggests that early diagnosis with complete imaging, laboratory tests, and histopathology can prevent complications and subsequently improve outcomes. Diagnostic protocols should be standardized to decrease variability in symptom duration and improve early detection. The authors identify some of the major gaps in the existing literature on diagnostic tools. Current investigative techniques, including CT and MRI, are of great value but poorly seem to distinguish O-TB from other orbital diseases. There is, therefore, a dire need to complement these methods of diagnosis with advanced molecular diagnostics to enhance accuracy, such as next-generation sequencing or improved PCR assays. A better understanding of the host immune response in O-TB constitutes another important area of research. The studies of immune factors and their relevance according to various age groups and different comorbidities may bring about the identification of biomarkers of disease severity and treatment response.

Finally, international guidelines on this global disease need to be taken into consideration with regional variation and health infrastructure. International collaborative studies may help in working out more effective management strategies with universal application.

## Conclusions

O-TB represents one of the rare, very critical diagnostic and therapeutic challenges in the discipline of neuro-ophthalmology, distinguished by its misleading clinical presentations mimicking other pathologies of the orbit after differentiation from within the given groups. This systematic review has focused on the role of comprehensive imaging modalities, including CT and MRI, combined with histopathological and molecular diagnostics in rendering an early and accurate diagnosis of O-TB. The efficacy of ATT in the management of O-TB is established, though the high incidence of complications like glaucoma and cataracts also brought out the need for careful long-term follow-up and surgical intervention when required.
